# High-Lard and High-Cholesterol Diet, but not High-Lard Diet, Leads to Metabolic Disorders in a Modified Dyslipidemia Model

**DOI:** 10.5935/abc.20190149

**Published:** 2019-11

**Authors:** Lidiane B. Muniz, Aline M. Alves-Santos, Fabricio Camargo, Danieli Brolo Martins, Mara Rubia N. Celes, Maria Margareth V. Naves

**Affiliations:** Universidade Federal de Goiás, Goiania, GO - Brazil

**Keywords:** Dietary Fats, Cholesterol, Lipid Metabolism, Fatty Liver, Inflammation, Rats

## Abstract

**Background:**

In view of the increased global prevalence of cardiovascular and hepatic diseases, the diet lipid content and its relationship with the accumulation of fat in hepatocytes have been investigated as key factors in preventing these diseases.

**Objective:**

To evaluate the metabolic effects of a high-lard diet supplemented or not with cholesterol on a modified dyslipidemia model.

**Methods:**

We divided 24 adult male Wistar rats into three groups: standard diet (STD - 4% lipids), high-lard diet (HLD - 21% lard), and high-lard and high-cholesterol diet (HL/HCD - 20% lard, 1% cholesterol, 0.1% cholic acid). After six weeks of treatment, blood and liver were collected for biochemical (serum lipid profile and liver enzymes) and morphological analyses. Statistical analysis included one-way analysis of variance (ANOVA), followed by Tukey test for mean comparisons, and a 5% probability was considered statistically significant.

**Results:**

Animals fed HL/HCD showed increased total cholesterol, triacylglycerol, LDL-c, non-HDL-c, alanine aminotransferase (ALT), and aspartate aminotransferase (AST) serum levels compared to those fed STD. In addition, the HL/HCD animals presented higher relative liver weight, with moderate macrovesicular hepatic steatosis and inflammatory infiltrate.

**Conclusion:**

A high-fat diet with lard (20%) and cholesterol (1%) triggered dyslipidemia with severe liver damage in rats in a shorter experimental time than the previously reported models. The high-lard diet without supplementation of cholesterol led to body weight gain, but not to dyslipidemia.

## Introduction

The worldwide prevalence of metabolic diseases and associated health complications have increased in recent decades. Excess body fat, insulin resistance, non-alcoholic fatty liver disease (NAFLD), and dyslipidemia lead to metabolic disorders related to higher risks of developing cardiovascular diseases.^1^ NAFLD ranges from a minor hepatocyte fat accumulation to more severe stages of inflammatory necrosis, with or without fibrosis. Studies indicate an association between NAFLD and insulin resistance, hepatocyte inflammation, and lipid metabolism impairment,^2,3^ as most NAFLD patients present dyslipidemia.^4^

Dyslipidemia is characterized by increased levels of triacylglycerol (TG) and low-density lipoprotein cholesterol (LDL-c) and decreased high-density lipoprotein cholesterol (HDL-c).^5^ Hypercholesterolemia (isolated LDL-c increase) and hypertriglyceridemia (isolated TG increase) are the most common forms of dyslipidemia.^6^ In general, lipid metabolism imbalance results from the interaction between genetics and environmental factors, such as eating habits, especially lipid consumption. Therefore, diet lipid content has been investigated as a key factor in preventing cardiovascular and hepatic diseases.^7^

Experimental studies with high-fat diets have used very high contents of dietary fat (about 50% of the diet composition), in short-period (four weeks) protocols, without finding serum lipid impairment.^8,9^ On the other hand, studies testing lipid concentrations closer to human consumption have been performed during a relatively long experimental time, as in previous reports with rats (21% fat for 9 weeks)^10^ and with mice (15% to 20% fat for 12 to 16 weeks).^11,12^

In this context, this study aimed to evaluate the metabolic effects of a high-lard diet supplemented or not with cholesterol on a modified dyslipidemia model, with shorter experimentation time and lower diet lipid concentration compared to previous reports.

## Methods

### Animals, diets, and experimental design

The experiment was carried out with adult male Wistar rats provided by the University of São Paulo (Ribeirão Preto, Brazil). The rats were kept in plastic cages (2 animals/cage) for seven weeks (one week of acclimation and six weeks of assay), under controlled environmental conditions (12 h light, 12 h dark cycle, and temperature at 22 ± 2ºC). In the experiment, we randomly divided 24 rats into 3 groups of 8 animals, according to a randomized block design based on their body weight. The sample size was defined considering previous studies on experimental dyslipidemia. Each group received one of the following diets: standard diet (STD; RH19521, Rhoster, Brazil) prepared according to AIN-93M,^13^ high-lard diet (HLD; RH195143, Rhoster, Brazil), and high-lard and high-cholesterol diet (HL/HCD; RH195142, Rhoster, Brazil). Soybean oil (STD) and lard were used as lipid sources; cholesterol, to induce hypercholesterolemia; and cholic acid, to increase the hypercholesterolemic effect of supplemented cholesterol^14^ (Table 1). The diets and filtered water were provided *ad libitum*. Dietary intake and body weight of the animals were monitored three times a week.

**Table 1:** Composition of the experimental diets

Component (g/100g of diet)		Diet^1^		
		STD	HLD	HL/HCD
Casein (82.93 g of protein)		16.47	16.47	16.47
L-cystine		0.18	0.18	0.18
Soybean oil		4.00	0.00	0.00
Lard^2^		0.00	21.00	20.00
Cholesterol^2^		0.00	0.00	1.00
Cholic acid^2^		0.00	0.00	0.10
Sucrose		10.00	10.00	10.00
Cellulose		5.00	5.00	5.00
Mineral mix		3.50	3.50	3.50
Choline bitartrate		0.25	0.25	0.25
Vitamin mix		1.00	1.00	1.00
*tert*-Butylhydroquinone (TBHQ)		0.008	0.008	0.008
Dextrinized cornstarch		15.50	15.50	15.50
Corn starch		44.09	27.09	26.99
Lipids (g/100g)^3^		4.1 ± 0.1	19.7 ± 1.4	20.8 ± 1.6
Energy from lipids^4^		36	189	189
Diet energy (kcal/g)^4^		3.41	4.26	4.26
(Energy from lipids x 100)/diet energy (%)		11	44	44

1 Reeves et al.^^13^^; STD: standard diet; HLD: high-lard diet; HL/HCD: high-lard and high-cholesterol diet;

2 Fernandes et al.^^10^^;

3 Result of diet chemical analysis;

4 Diet energy value: 4, 4, and 9 kcal/g for protein, carbohydrate, and lipid, respectively.

After six weeks of experimentation, the animals were euthanized (xylazine + ketamine/ 10 mg/kg + 100 mg/kg). Next, we collected blood samples by abdominal puncture (about 5 mL) for biochemical analyses (serum lipid profile and liver enzymes). After the euthanasia by abdominal aorta exsanguination, we extracted, weighed, and prepared the liver of the animals for morphological evaluation. All procedures complied with the Guide for the Care and Use of Laboratory Animals^15^ and were approved by the Animal Care and Use Ethics Committee of the Federal University of Goiás (protocol number 039/15).

### Biochemical analyses

Blood samples were collected in heparin tubes and centrifuged at 4000 rpm for 10 min to separate the serum, which was immediately stored at -80°C for analyses of lipid profile and liver enzymes. Total cholesterol (TC), TG, LDL-c, HDL-c, alanine aminotransferase (ALT), and aspartate aminotransferase (AST) levels were determined by commercially available kits (Labtest Diagnóstica S.A., Lagoa Santa, Brazil). Very low-density lipoprotein cholesterol (VLDL-c) was estimated by the Friedewald equation, and non-HDL-c fraction was obtained by the difference between TC and HDL-c levels. The importance of non-HDL-c fraction has been highlighted in cardiovascular disease (CVD) risk prediction. Boekholdt et al.^16^ revealed that non-HDL-c concentrations had a stronger direct association with CVD risk than LDL-c and apolipoprotein B (Apo B).

### Liver morphological evaluation

We rinsed the liver lobes in ice-cold 0.9% physiological solution and fixed them by immersion in phosphate-buffered 10% formalin for 24 h. Subsequently, the lobes were cut into 4 to 5 mm thick fragments, dehydrated in increasing alcohol concentrations (80%, 95%, and 100%), clarified in xylol (3 baths of 30 min each), and embedded in paraffin. Five-micrometer-thick serial sections were obtained and stained with hematoxylin and eosin (HE). We used the HE-stained sections to analyze the alterations in the arrangement of hepatic parenchyma and the presence of inflammatory infiltrates with a Leica Las V4 Software (Leica Imaging Systems Ltd., Cambridge, UK), a Leica DM2000 microscope (Leica Microsystems Wetzlar GmbH, Wetzlar, Switzerland), a Leica DC230 video camera (Leica Microsystems AG, Heerbrugg, Switzerland), and an online computer. Twenty fields of hepatic parenchyma of each animal were randomly selected and analyzed, at 100x and 400x magnification.

### Statistical analysis

Data were expressed as mean ± standard deviation. The literature considers body weight and biochemical parameters obtained in animal models as parametric data.^8-12^ Thus, we determined statistical significance using the one-way analysis of variance (ANOVA), followed by Tukey test for mean comparisons (p < 0.05). STATISTICA software, version 7.0 (StatSoft, Inc., Tulsa, OK, USA) was used for statistical analyses.

## Results

The lard used in the formulation of diets contained 39 g/100 g of saturated fat and 72 mg/100 g of cholesterol. In high-fat diets (HLD and HL/HCD), lard contributed with approximately 15 mg of cholesterol/100 g of diet, besides the cholesterol added to the HL/HCD. Forty-four percent (44%) of the HLD and HL/HCD energy derived from lipids, a value four times higher than that of STD (11%).

The initial body weight of the animals did not differ among the groups, ensuring the homogeneity of group repetitions. Animals fed HLD showed higher body weight gain than those from the STD group. Liver weight and relative liver weight of animals fed HL/HCD were higher (p < 0.05) than those of other groups (Table 2).

**Table 2:** Body and liver weight and biochemical parameters of Wistar rats after six weeks of treatment with standard and high-fat diets

Parameter	Diet		
	STD	HLD	HL/HCD
**Body weight (g)**			
Initial	249.05 ± 21.44^a^	243.85 ± 15.09^a^	242.35 ± 16.78^a^
Final	499.58 ± 65.32^a^	564.08 ± 39.73^a^	508.58 ± 47.68^a^
Gain	250.53 ± 48.40^b^	320.23 ± 44.50^a^	266.23 ± 38.21^a,b^
**Liver**			
Weight (g)	14.90 ± 2.33^b^	15.92 ± 1.65^b^	27.10 ± 5.94^a^
Relative weight (g/100g body weight)	2.98 ± 0.24^b^	2.82 ± 0.14^b^	5.28 ± 0.80^a^
**Serum lipid profile (mg/dL)**			
TC	59.83 ± 15.99^b^	64.58 ± 14.13^b^	87.75 ± 8.54^a^
LDL-c	4.23 ± 0.71^b^	6.98 ± 2.09^b^	23.63 ± 4.55^a^
HDL-c	30.74 ± 4.11^a^	34.42 ± 7.77^a^	26.74 ± 4.23^a^
non-HDL-c	29.09 ± 12.13^b^	30.17 ± 10.84^b^	61.01 ± 6.95^a^
VLDL-c	10.68 ± 1.58^b^	16.77 ± 4.58^a,b^	18.52 ± 7.51^a^
TG	53.42 ± 7.91^b^	83.83 ± 22.90^a,b^	92.58 ± 37.55^a^
**Liver enzymes (U/L)**			
ALT	48.72 ± 15.91^b^	28.16 ± 6.00^b^	210.30 ± 137.78^a^
AST	154.67 ± 22.42^b^	117.00 ± 29.84^b^	300.63 ± 60.26^a^

Data are expressed as mean ± standard deviation. a,bValues in the same row with different superscript letters are significantly different (Tukey test, p < 0.05). STD: standard diet; HLD: high-lard diet; HL/HCD: high-lard and high-cholesterol diet; Relative liver weight = (liver weight/body weight) x 100; TC: total cholesterol; LDL-c: low-density lipoprotein cholesterol; HDL-c: high-density lipoprotein cholesterol; VLDL-c: very low-density lipoprotein cholesterol; TG: triacylglycerol; non-HDL-c: non-high-density lipoprotein cholesterol; ALT: alanine aminotransferase; AST: aspartate aminotransferase.

Animals fed HL/HCD showed higher TC, TG, LDL-c, VLDL-c, non-HDL-c, ALT, and AST serum levels than those from STD and HLD groups. Serum lipid profile and liver enzymes of animals fed HLD were not different from those of the STD group (Table 2).

**Figure 1:**
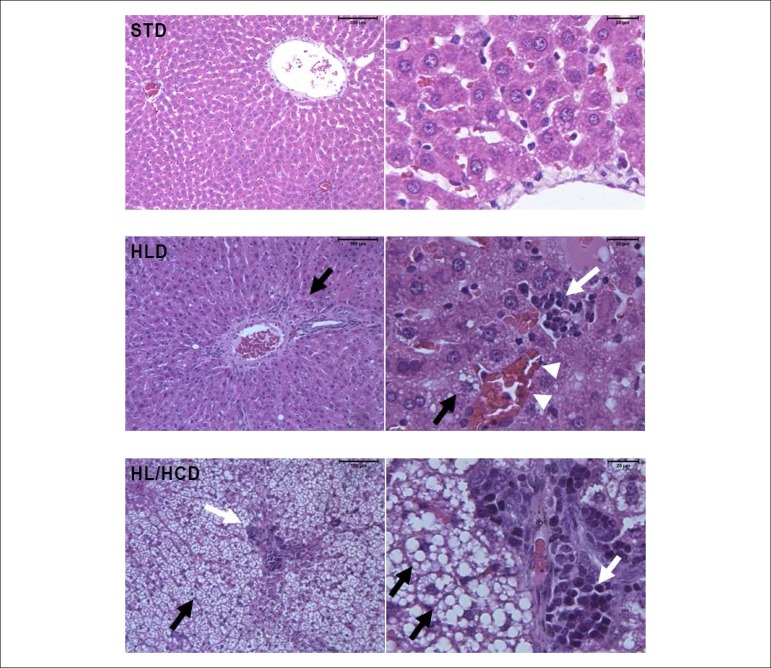
Morphological evaluation of the liver of Wistar rats fed high-fat diets for six weeks. STD (standard diet): preserved hepatic tissue architecture with conserved vascular distribution. HLD (high-lard diet): preserved hepatic tissue architecture with mild and uniform hepatic steatosis (black arrows), mild inflammatory infiltrate (white arrows), and mild passive hyperemia (arrowheads). HL/HCD (high-lard and high-cholesterol diet): altered tissue architecture with moderate hepatic steatosis, fat microvesicles and macrovesicles (black arrows), and marked inflammatory infiltrate (white arrows). The bars indicate 100 µm at 100x magnification (left panel) and 20 µm at 400x magnification (right panel).

We found microscopic changes (Figure 1) in the liver of animals fed HL/HCD, as the hepatic tissue architecture was not preserved, with hepatocyte ballooning (characterized by swelling and/or vacuolation), alterations in sinusoidal blood capillaries, and passive hyperemia (capillaries and veins engorged with blood). In addition, rats fed HL/HCD presented moderate macrovesicular hepatic steatosis associated with a marked infiltration by lymphomononuclear cells. On the other hand, animals fed HLD had their hepatic tissue architecture preserved, containing hepatocytes with usual morphological aspect, and preserved vascular distribution, centrilobular vein, and portal triad. Furthermore, the HLD group showed mild microvesicular hepatic steatosis associated with lower infiltration by lymphomononuclear cells. The hepatic tissue architecture of the STD group was maintained with typical hepatocytes, preserved hepatic sinusoid, and capillary strands; conserved vascular distribution, intact portal triads, with portal vein branches, hepatic artery, and bile duct.

## Discussion

Dyslipidemia was triggered in rats fed a diet with 20% lard and 1% cholesterol, in six weeks, with increased liver weight, liver injury, and inflammatory infiltrate.

In the present study, lipids contributed with 44% of high-fat diet energy, a percentage close to that reported for high-fat diet consumed by overweight or obese individuals (43% to 55%).^17-19^ In previous animal studies, lipid contribution ranged from 67% to 75% of high-fat diet energy.^8,9^ Therefore, these diets with very high contents of lipids do not reproduce, realistically, the high-fat diets consumed by humans. The daily recommendation for human adults is 20% to 35% energy from lipids, according to the AMDR (Acceptable Macronutrient Distribution Ranges), considering that high lipid intake indicates a greater risk of obesity and associated disorders.^20^

Higher body weight gain in the HLD group can be explained by its higher food consumption (17.6 g/day/rat) compared to the HL/HCD group (13.8 g/day/rat) since the energy density of the diets was similar (4.26 kcal/g). Higher weight gain was reported in a previous study with mice fed a high-fat and high-cholesterol diet (energy density = 4.27 kcal/g) for 12 weeks, compared to the control group.^12^

Despite the higher body weight gain, the HLD group did not differ from the STD group regarding liver weight (Table 2). The relative liver weight in the HL/HCD group was very high, above 5% of the body weight of these animals. The increased liver weight in the HL/HCD group is related to the strong presence of fat vesicles in hepatocytes, as observed in the liver morphological evaluation (Figure 1). A previous study with mice on a high-fat and high-cholesterol diet, without cholic acid, for 16 weeks presented similar results regarding liver weight and hepatic tissue lipid accumulation.^11^ In our study, the cholic acid probably accelerated the dyslipidemic effects of cholesterol in the HL/HCD group, given that we identified metabolic disorders in only 6 weeks, whereas the study by Jung et al.^11^ reached the same effects in 16 weeks. The serum lipid impairment observed in animals fed HL/HCD is characteristic of dyslipidemia, and the marked increase in LDL-c may be related to the reduction in LDL-c receptor activity in hepatocytes. Previous studies revealed changes in TC and TG serum levels of rats and mice fed a high-fat and high-cholesterol diet.^10,12^ However, the experimentation periods of the reported studies (9 and 12 weeks, respectively) were longer than that used in the present study.

The serum lipid profile of the animals fed HLD did not differ from those fed STD. Studies with Wistar rats on 50% to 55% lard diets (67% to 75% of the diet energy) for four weeks showed similar results.^8,9^ The harmful effect of very high intake of saturated fatty acids and its relation to dyslipidemia have been questioned.^1^ Siri et al.^21^ highlighted that the effects of saturated fat on serum lipid profile could be modulated by the content and/or availability of polyunsaturated fatty acids, so that saturated fat would only increase LDL-c if the polyunsaturated fat intake is below a threshold level (5% of the diet energy).^22^ Our results confirmed that the excessive intake of saturated fatty acids explains only part of the changes in serum lipid profile. Other factors influence these changes, such as dietary cholesterol, obesity, insulin resistance, and hypertriglyceridemia.^23,24^

High ALT and AST serum levels are characteristic of NAFLD and are associated with insulin resistance. In this study, increased ALT and AST serum levels were compatible with the hepatic damages observed in the animals fed HL/HCD. Other studies also found increased ALT and AST serum levels in mice fed a high-fat and high-cholesterol diet.^11,12^

The morphological evaluation showed marked changes in the hepatic tissue of animals fed HL/HCD, such as fatty macrovesicles and inflammatory infiltrates, characterizing a moderate hepatic steatosis,^25^ which was confirmed by the results of the hepatic enzymes (ALT and AST). Fat accumulation in the liver of these animals is related to high cholesterol consumption, as this overload in the cells alters the cholesterol homeostasis.^26^ In addition, the accumulation of intermediate lipid metabolites, such as diacylglycerol and acylcarnitines, is associated with inflammation and insulin resistance.^27,28^ A previous study suggested that high dietary cholesterol is a critical factor for the progression of hepatic steatosis and inflammation in animal models. However, features of NAFLD are more evident when the supplemented cholesterol is associated with cholic acid in a high-fat diet, as observed in our study.

The presence of inflammatory infiltrate in the hepatic tissue promotes cytokine and chemokine secretion, such as tumor necrosis factor a (TNF-a) and interleukin 6 (IL-6), which induce insulin resistance. In this metabolic disorder, the increased lipolysis of TG stored in the adipose tissue raises fatty acid production. The fatty acids released in the circulation, in turn, inhibit the anti-lipolytic action of insulin and promote higher lipid absorption by the liver, leading to dyslipidemia and hepatic steatosis.^3,32,33^ Hamsters on a 10-week hyperlipidemic diet containing cholesterol (0.2%) developed dyslipidemia and hepatic steatosis, with impaired TC, TG, LDL-c, ALT, and AST serum concentrations.^34^ Liver histopathological analysis of Sprague-Dawley rats fed a high-cholesterol diet (1.5%) also revealed hepatic steatosis and inflammation.^35^

One limitation of our study was the impossibility to use more accurate methods to evaluate liver fat macro- and microvesicles and foam cells, such as Oil Red and immunohistochemistry, respectively, which could allow us to analyze liver damages quantitatively. Moreover, we recommend further studies, including a treatment with lard and cholic acid, to investigate whether cholic acid can enhance the metabolic effects of lard cholesterol.

## Conclusion

A high-fat diet with lard (20%) and cholesterol (1%) triggered dyslipidemia with severe liver damage in rats in a shorter experimental time than the previously reported models. The high-lard diet without supplementation of cholesterol led to body weight gain, but not to dyslipidemia. This model may be useful to investigate metabolic disorders in different experimental designs related to dyslipidemia and its comorbidities.
